# Characterization of the Oral Microbiota of Captive Viperid Snakes in Panama: Implications for Snakebite-Associated Wound Infections

**DOI:** 10.3390/toxins18100440

**Published:** 2026-10-13

**Authors:** Stephany Young Yusty, Celestino Aguilar, Alexa Prescilla-Ledezma, Alexander A. Martínez, Claudia González, Hildaura Acosta de Patiño

**Affiliations:** 1 Departamento de Microbiología Humana, Facultad de Medicina, Universidad de Panamá, Panama City 0816-00588, Panama; 2 Departamento de Investigación en Genómica y Proteómica, Instituto Conmemorativo Gorgas de Estudios de la Salud, Panama City 0816-02593, Panama; 3 Centro de Investigación e Información de Medicamentos y Tóxicos (CIIMET), Universidad de Panamá, Panama City 0816-00588, Panama

**Keywords:** snake oral microbiota, 16S rRNA sequencing, viperid snakes, snakebite-associated infection, Panama

## Abstract

Snakebite envenoming is a major neglected public health problem in Panama, yet culture-independent data on the oral microbiota of medically important viperid snakes remain limited. We characterized the oral microbiota of four captive species native to Panama (*Bothrops asper*, *Bothriechis nigroviridis*, *Cerrophidion sasai*, and *Porthidium lansbergii*) using forward-read analysis of 16S rRNA V3–V4 amplicons and compositional methods. Among 68 retained oral samples, communities were dominated by Pseudomonadota, Bacillota, Actinomycetota, and Bacteroidota. Observed ASV richness differed among species (Kruskal–Wallis H = 21.87, *p* < 0.001), whereas no significant differences were detected in Shannon diversity or Pielou’s evenness. Richness differences require caution because sequenced negative controls were unavailable and no independent depth-aware sensitivity analysis was performed. Community composition was significantly associated with host species (PERMANOVA R^2^ = 0.197, *p* = 0.001), although PERMDISP detected heterogeneous within-group dispersion (*p* = 0.005), requiring cautious interpretation. ANCOM-BC2 identified 21 differentially abundant genera, including *Providencia*, *Morganella*, *Pseudomonas*, *Stenotrophomonas*, and *Staphylococcus*, with high relative abundance of *Mycoplasma* in a subset of *P. lansbergii* samples. These findings provide a culture-independent taxonomic baseline for future culture-based, antimicrobial-susceptibility, and paired snake-oral/human-wound investigations in Panama.

## 1. Introduction

Venomous snakebites are a major global public health problem, especially in tropical and subtropical regions where incidence and mortality are highest [1,2]. Snakebite envenoming causes an estimated 80,000–130,000 deaths annually and leaves ~400,000 survivors with permanent neurological, musculoskeletal or psychological sequelae, a disease burden concentrated in tropical and subtropical regions [1,3,4,5,6,7,8,9]. Clinical manifestations of snakebites vary widely and depend on multiple factors, including the snake species, the volume of venom injected, and how quickly appropriate medical treatment is administered [7,8,10]. Snake venoms contain a complex mix of neurotoxins, hemotoxins, and cytotoxins that can cause symptoms ranging from local pain and swelling to systemic manifestations, including shock, paralysis, and renal failure [11,12,13,14,15,16,17]. Snakebite wounds may be exposed to bacteria derived from the snake oral microbiota, as well as from environmental and skin-associated sources [18,19,20,21,22]. Recent studies have linked diverse bacterial species to snakebite infections, emphasizing the importance of understanding these complications for improved patient management [9,23,24,25,26]. Culture-based studies have recovered potentially pathogenic taxa from snake oral samples, including *Morganella morganii*, *Pseudomonas aeruginosa*, and *Enterobacter* spp., which have also been associated with snakebite-associated wound infections [9,23,27,28,29,30]. Microbiota studies are fundamental to understanding host health and offer insights into key evolutionary processes such as coevolution, adaptation, and antibiotic resistance emergence [31,32]. Prior work has indicated that snake oral microbiotas can differ substantially depending on species identity, time of year, habitat type, health status, and feeding or predatory behavior [27,33,34,35].

Few studies have characterized the oral microbiota of venomous snakes using culture-independent high-throughput sequencing, and this gap is particularly pronounced in Latin America, where available data remain largely culture-based and restricted to specific taxa or clinical contexts like snakebite wound infections [25,28,29,30,36,37,38]. Sequencing-based studies from India, Taiwan, China, and the Philippines have described diverse snake oral bacterial communities that vary among host species and include genera containing clinically relevant taxa [9,29,30,36]. Culture-independent sequencing data on the oral microbiota of medically important venomous snakes in Panama remain limited, despite this country bearing one of the highest incidence rates of snakebite in Central America (approximately 34–65 cases per 100,000 inhabitants), a burden attributed mainly to *Bothrops asper* [39]. Panama has been repeatedly highlighted as a hotspot where neglected tropical diseases, including snakebite envenoming, disproportionately affect impoverished rural and indigenous communities with limited access to timely medical care and antivenom [39,40,41,42,43,44,45].

Using 16S rRNA amplicon sequencing targeting the V3–V4 region, the present study provides a culture-independent characterization of the oral microbiota of captive medically important viperid snakes in Panama. Although culture-based studies from Panama have begun to characterize snake oral bacteria and antimicrobial susceptibility patterns, culture-independent data remain limited, and direct microbiological linkage between snake oral communities and infected human bite wounds has not yet been established [22]. The present study therefore provides a complementary taxonomic baseline intended to inform, rather than substitute for, culture-based diagnostics and antimicrobial susceptibility testing.

## 2. Results

### 2.1. Sequencing Depth, Quality Filtering, and Dataset Composition

A total of 73 biological specimens were represented in the initial sequencing dataset, comprising 68 oral swabs and five venom specimens. Because the present study focused on the oral microbiota, the five venom specimens were excluded from downstream oral-community analyses, resulting in a dataset of 68 oral samples. The ASV feature-table dimension remained 3446 after restriction to the oral samples. Removal of mitochondrial and chloroplast-associated features reduced the dataset to 3235 ASVs. Subsequent prevalence–abundance filtering, retaining ASVs detected in ≥5% of samples with ≥10 reads, yielded a final analytical dataset of 300 ASVs for downstream statistical analyses (Table 1). Sequencing depth across the 68 oral samples ranged from 49,273 to 228,164 reads (species-specific ranges are provided in Table 2), and rarefaction was performed at 49,273 reads/sample, corresponding to the minimum sequencing depth observed among the 68 retained samples. An outlier threshold was defined a priori as 10% of the median read count (11,612 reads); no samples fell below this threshold, and all 68 samples were therefore retained for rarefaction (Figure 1).

### 2.2. Sequencing Adequacy

Good’s coverage estimates were uniformly high across host species (mean range: 0.9998–1.0000; Supplementary Table S1a) and are reported descriptively as an indicator of sequencing coverage. Because denoising and subsequent abundance/prevalence filtering alter the low-frequency portion of the ASV distribution, Good’s coverage was not subjected to hypothesis testing. Chao1 and ACE were likewise not reported because these estimators depend strongly on low-frequency taxa; observed ASV richness was therefore retained as the primary richness measure. These coverage estimates describe the analysed ASV table and should not be interpreted as evidence that the complete oral bacterial community was exhaustively sampled.

### 2.3. Alpha Diversity Across Host Species

Observed ASV richness differed significantly among the four viperid species (Kruskal–Wallis, H = 21.87, df = 3, *p* < 0.001, *n* = 68) (Figure 2). Post hoc Dunn’s tests with Benjamini–Hochberg correction showed significantly higher richness in *B. nigroviridis* than in *B. asper* (Z = −3.746, adjusted *p* < 0.001) and *P. lansbergii* (Z = −4.423, adjusted *p* < 0.001); all other pairwise comparisons were non-significant after correction (adjusted *p* > 0.05; Supplementary Table S1c). Shannon diversity (H = 7.07, df = 3, *p* = 0.070) and Pielou’s evenness (H = 4.40, df = 3, *p* = 0.221) did not differ significantly among host species (Supplementary Table S1b).

### 2.4. Taxonomic Composition

At the phylum level, oral communities across all four host species were dominated by Pseudomonadota (mean relative abundance 47.9–87.3% depending on species), with variable secondary contributions from Bacillota (3.3–30.5%), Actinomycetota (3.5–17.8%), and Bacteroidota (0.1–5.8%) (Figure 3; Supplementary Table S3). *Cerrophidion sasai* and *P. lansbergii* showed comparatively lower Pseudomonadota dominance and correspondingly higher Bacillota and Actinomycetota contributions relative to *B. asper* and *B. nigroviridis*, consistent with species-specific compositional differences within a broadly conserved phylum-level structure.

At the genus level, the 20 most abundant genera (Figure 4; Table 3) revealed pronounced host species-associated patterns. *Morganella* and *Providencia* were detected across all four host species, with mean relative abundances of 5.29–21.32% and 13.96–31.09%, respectively. These genera contain clinically relevant taxa, but the present genus-level assignments do not establish the presence of pathogenic species or strains. *Pseudomonas* was likewise abundant across all species, reaching its highest mean relative abundance in *B. nigroviridis* (35.01%), while *Stenotrophomonas* showed a parallel pattern of elevated abundance in the same species. *Mycoplasma* was detected in 26 of 30 *P. lansbergii* samples (86.7%), with a strongly right-skewed relative abundance distribution (mean 15.35%, median 0.06%, SD 28.00%). The elevated mean was driven by a subset of samples and did not indicate uniformly high relative abundance across individuals. In each of the other three host species, the mean relative abundance was 0.01%.

### 2.5. Beta Diversity and Community Structure

Beta diversity, evaluated using Aitchison distances on CLR-transformed genus-level counts, showed a statistically significant association between host species and oral community composition (PERMANOVA/adonis2, F = 5.242, R^2^ = 0.197, *p* = 0.001, 999 permutations; Figure 5). The first two principal coordinates represented 10.6% and 7.3% of the total variation, respectively, accounting for 17.9% together. PERMDISP indicated significant heterogeneity in multivariate dispersion across host species (F = 4.992, *p* = 0.005), indicating that within-group variance was not uniform among species. Pairwise PERMDISP comparisons (Tukey HSD) indicated that this dispersion heterogeneity was driven primarily by the contrast between *P. lansbergii* and *B. asper* (adjusted *p* = 0.0016), while no other pairwise comparison reached significance (adjusted *p* > 0.05). Given the heterogeneous dispersion and unequal group sizes, the global PERMANOVA result should be interpreted cautiously. The non-significant pairwise PERMDISP comparisons do not establish homogeneous dispersion in the remaining groups, and the analysis does not provide unequivocal evidence of separation between group centroids. Ordination showed that *B. nigroviridis* and *B. asper* occupied largely distinct regions of compositional space, whereas *C. sasai* and *P. lansbergii* samples showed substantial overlap.

### 2.6. Differential Abundance of Bacterial Genera Across Host Species

Differential abundance analysis using ANCOM-BC2 with Holm–Bonferroni correction (global test across all four host species) identified 21 genera with statistically significant differential abundance (q < 0.05; Figure 6, Supplementary Table S2). These included *Providencia*, *Morganella*, *Pseudomonas*, *Stenotrophomonas*, and *Staphylococcus*, genera containing taxa previously implicated in snakebite-associated wound infections [9,25]. Hierarchical clustering of mean CLR-transformed abundances (Figure 6) grouped *Providencia*, *Morganella*, *Pseudomonas*, and *Stenotrophomonas* together as consistently high-abundance taxa across most species, whereas *Mycoplasma* clustered distinctly, reflecting its distinct host-associated abundance pattern in *P. lansbergii*. This differential abundance pattern further supports an association between host species and oral microbiota composition and identifies several genera containing taxa of potential clinical relevance that warrant targeted culture-based validation.

## 3. Discussion

The oral cavity of viperid snakes is a plausible reservoir of opportunistic bacteria implicated in post-envenomation wound infection [9,22]. Culture-independent characterizations, however, remain scarce for Central America [46]. This study provides a quantitatively grounded description of the oral bacterial microbiota of four medically important viperid species native to Panama, in which host species was significantly associated with oral microbiota composition, alongside a defined panel of genera of potential clinical relevance.

### 3.1. Host Species and Microbial Richness

Host species was significantly associated with observed ASV richness, whereas Shannon diversity and Pielou’s evenness did not differ significantly (Figure 2; Section 2.3). *B. nigroviridis* showed the highest richness, with significant differences relative to *B. asper* and *P. lansbergii* after multiple-comparison correction. These results indicate host-associated differences in the number of detectable ASVs without corresponding significant differences in overall diversity or evenness [47,48].

Beta-diversity analysis identified an association between host species and oral community composition (Figure 5). However, significant PERMDISP heterogeneity and unequal group sizes limit interpretation of the PERMANOVA result as unequivocal evidence of differences in group centroids. In the PCoA, *C. sasai* and *P. lansbergii* showed substantial overlap, whereas *B. nigroviridis* and *B. asper* occupied largely distinct regions of compositional space. Methodological choices also affect the interpretation of microbiota comparisons, including the estimation of alpha diversity [49] and the selection of differential-abundance methods [50].

Variation in host traits, including body size, and environmental exposures has been associated with differences in microbial communities across vertebrates [47,48,51]. These factors may contribute to the richness patterns observed here, although their individual effects were not evaluated. The snakes were maintained under controlled husbandry conditions, but their diets were adjusted to species and body size, and specimens were housed at two facilities. The present design therefore cannot separate host-species associations from differences in husbandry, facility, or other unmeasured exposures. Studies of captive reptiles likewise indicate that both host-associated and environmental factors can contribute to microbiota variation [47,52].

### 3.2. Taxonomic Architecture and Genera of Potential Clinical Relevance

Pseudomonadota predominated across the four host species, broadly consistent with previous sequencing-based studies of snake oral communities [28,29,30]. This similarity indicates a shared broad taxonomic pattern, but does not establish equivalent species composition, ecological function, or pathogenic potential. In the present dataset, *Morganella*, *Providencia*, and *Pseudomonas* contributed substantially to this predominance. Host-associated conditions and captive environmental exposures are possible contributors to these profiles, although their respective effects were not evaluated directly. *Morganella* and *Providencia* were detected in all four host species, consistent with previous culture- and sequencing-based observations in venomous snakes [29,53,54]. Because all sampled snakes met the study’s apparent-health criteria, detection of *Morganella* is compatible with oral carriage in apparently healthy captive individuals; however, the present cross-sectional DNA-based design cannot distinguish stable oral residence from transient acquisition through diet, husbandry, or environmental exposure. Their detection across host species did not imply uniform relative abundance among individuals, as illustrated by the substantial inter-individual variability shown in Figure 4 and Table 3. *Pseudomonas* and *Stenotrophomonas* reached their highest relative abundances in *B. nigroviridis* while remaining detectable at lower levels in the other species, consistent with host-associated quantitative differences within a broadly shared set of bacterial genera [29,47].

These findings are broadly consistent with prior Panamanian characterizations of viperid oral microbiota [46,55]. An earlier culture- and fingerprinting-based study of *B. asper* oral and venom bacterial communities reported *Morganella morganii* and *Providencia rettgeri* among the most frequently isolated taxa [55]. A more recent culture-dependent comparison of the same four species examined here likewise identified *M. morganii* and *P. rettgeri* as the predominant Gram-negative isolates, with *P. lansbergii* showing the highest bacterial diversity by culture-dependent methods [46]. This contrasts with the elevated ASV richness observed for *B. nigroviridis* in the present culture-independent dataset, which may reflect differences in detection methods, sampled individuals, or study conditions; the comparison does not isolate the contribution of each factor [46]. Notably, that same study reported antimicrobial susceptibility data not available from the present amplicon-based approach, including high resistance rates to first-generation cephalosporins in *M. morganii* and emerging extended-spectrum beta-lactamase production in *Enterobacter cloacae*. These findings reinforce the potential clinical relevance of the shared genera identified here and underscore the complementarity between culture-independent taxonomic profiling and culture-based susceptibility testing in Panama [46].

*Mycoplasma*-assigned sequences were detected in 26 of 30 *P. lansbergii* samples (86.7%). The mean relative abundance was 15.35%, whereas the median was 0.06% (SD 28.00%), indicating that high relative abundance in a subset of samples strongly influenced the mean. ANCOM-BC2 identified a global difference among host species (q = 0.00001), although this result does not establish each individual pairwise contrast. *Mycoplasma* has also been detected in oral samples of other snake species [36]. Reports of *Mycoplasma*-associated respiratory disease in tortoises [56] cannot be extrapolated to the genus-level detections in the apparently healthy snakes examined here. Taxonomic assignments can be influenced by the reference database used [57]. Experimental evidence from other animal models also indicates that host-associated factors can influence microbiota assembly [58], although this mechanism was not tested in the present study. The cross-sectional design cannot distinguish stable colonization, transient acquisition, or subclinical infection, and relative abundance does not measure absolute bacterial load. The partial 16S sequences and absence of sequenced negative controls further limit interpretation. These findings warrant targeted confirmation but do not establish pathogenicity to snakes or humans.

### 3.3. Genera of Potential Clinical Relevance and Antimicrobial Stewardship Considerations

ANCOM-BC2 identified 21 genera with significant differential abundance across host species, including genera containing taxa previously associated with wound infections (*Providencia*, *Morganella*, *Pseudomonas*, *Staphylococcus*, *Stenotrophomonas*) alongside skin- and environment-associated genera (*Cutibacterium*, *Corynebacterium*, *Rhodococcus*, *Brevibacterium*). Hierarchical clustering (Figure 6) grouped *Providencia*, *Morganella*, *Pseudomonas*, and *Stenotrophomonas* as a consistently high-abundance cluster, while *Mycoplasma* segregated distinctly, reflecting its comparatively high relative abundance in a subset of *P. lansbergii* samples. The same interpretive limitation applies to *Pseudomonas*, *Staphylococcus*, and *Stenotrophomonas*: their detection identifies genera containing clinically relevant taxa, without confirming pathogenic species, viable organisms, or infection.

Because 16S rRNA profiling establishes taxonomic signatures but does not demonstrate bacterial viability, antimicrobial susceptibility, or resistance determinants, the present findings should be considered hypothesis-generating rather than treatment-prescriptive [59,60]. The detection of genera that may include taxa with clinically relevant resistance mechanisms should therefore not be interpreted as evidence of resistance in the present samples [18,61]. Culture and antimicrobial susceptibility testing should be prioritized in clinically infected wounds whenever feasible [9,62]. The taxa identified here may help prioritize organisms for prospective culture-based investigation but should not, by themselves, guide organism- or drug-specific empirical therapy. This interpretation is consistent with randomized clinical trial evidence showing that routine preemptive chloramphenicol or amoxicillin–clavulanate prophylaxis did not significantly reduce secondary wound infection following *Bothrops* envenomation [63,64].

Taken together, these findings indicate host-associated differences in richness and community composition, the presence of shared genera of potential clinical relevance, and a distinct host-associated abundance pattern for *Mycoplasma* in *P. lansbergii*. These patterns offer an initial basis for prioritizing taxa warranting future culture-based validation in Panama.

### 3.4. Methodological Considerations and Study Limitations

Several factors qualify these findings. As noted above, PERMDISP heterogeneity tempers interpretation of the PCoA centroid separation. All specimens were captive, and captivity can alter microbial communities [52,65,66]. The findings therefore describe this captive collection and cannot be assumed to represent wild conspecifics. Husbandry and facility-related exposures may also contribute to the observed differences among host species groups. Although the PCR target spanned the approximately 460-bp V3–V4 region, taxonomic inference was based on an approximately 233-bp forward-read segment retained after primer removal. The reduced sequence length relative to the complete V3–V4 amplicon may limit discrimination among closely related taxa, particularly at the species and strain levels; accordingly, genus-level assignments reported here should not be interpreted as confirmation of pathogenic species or strains [59,67,68]. The absence of extraction and library negative controls precluded control-based contaminant identification and formal decontamination [69,70]. Retrospective screening cannot quantify or exclude reagent-derived contamination. Such contamination may alter taxonomic proportions, introduce low-abundance ASVs, and influence observed richness [69,70,71]. Sequencing depth can also affect microbial detection [72]. In the present dataset, observed richness was weakly positively correlated with sequencing depth (Spearman ρ = 0.253, *p* = 0.038), indicating that depth-related detection effects cannot be excluded. This correlation does not constitute a test of contamination.

Notably, *Cutibacterium*, a genus frequently reported as a reagent- and extraction-kit contaminant [69], was detected in nearly all samples and reached a mean relative abundance of 4.83% in *C. sasai* (Supplementary Table S4). It was also identified as differentially abundant by ANCOM-BC2 (q < 0.0001). In the absence of sequenced extraction and library negative controls, a contribution from differential contamination cannot be excluded; therefore, the *Cutibacterium* signal should be interpreted cautiously and requires confirmation using targeted molecular and/or culture-based approaches.

No independent depth-aware sensitivity analysis was performed; therefore, consistency of the alpha-diversity conclusions with an alternative approach remains unestablished. This limitation is particularly relevant because observed richness was the only alpha-diversity metric showing significant differences among species. The richness differences should consequently be interpreted as exploratory, considering the potential effects of contamination, filtering, and depth-related detection [49,70,73]. In addition, sample sizes were unbalanced across host species because of captive availability, potentially reducing statistical power—particularly for comparisons involving less-represented species—and increasing uncertainty around effect estimates [74,75]. Finally, the cross-sectional design precluded assessment of temporal stability and within-host microbial dynamics. Moreover, DNA-based 16S rRNA amplicon sequencing provides no direct evidence of bacterial viability, antimicrobial resistance genes, or functional capacity [59,60,76]. Future longitudinal studies should therefore combine near-full-length 16S rRNA sequencing or shotgun metagenomics with culture-based isolation, phenotypic antimicrobial susceptibility testing, and paired sampling of snake oral communities and clinically infected human bite wounds.

## 4. Conclusions

This study provides a statistically grounded characterization of the oral microbiota of four medically important viperid species (*Bothrops asper*, *Bothriechis nigroviridis*, *Cerrophidion sasai*, and *Porthidium lansbergii*) maintained at the CIIMET serpentarium in Panama. Across 68 retained oral samples, bacterial communities were dominated by Pseudomonadota, Bacillota, Actinomycetota, and Bacteroidota. Observed ASV richness differed among host species, and community composition was significantly associated with host species, although PERMDISP indicated unequal within-group dispersion that warrants cautious interpretation of compositional separation. *Morganella*, *Providencia*, and *Pseudomonas* were detected across all four host species, while *Mycoplasma* showed comparatively high relative abundance in a subset of *P. lansbergii* samples. The contribution of contamination could not be quantified because sequenced extraction and library negative controls were unavailable. Richness differences should therefore be interpreted cautiously, particularly because their robustness to an alternative depth-aware analysis was not established. Collectively, these findings establish a culture-independent taxonomic baseline for captive viperid oral communities in Panama and support prospective studies combining higher-resolution sequencing with culture-based isolation, antimicrobial susceptibility testing, and paired sampling of snake oral communities and infected human bite wounds.

## 5. Materials and Methods

### 5.1. Study Design and Eligibility Criteria

This descriptive, cross-sectional study characterized the oral bacterial diversity of venomous snakes using next-generation sequencing, with samples collected at a single time point. The study population comprised venomous snake specimens housed at the serpentarium of the Centro de Investigación e Información de Medicamentos y Tóxicos (CIIMET), at the Octavio Méndez Pereira and Santiago de Veraguas facilities. Eligible specimens were selected through a non-probabilistic, convenience sampling approach based on predefined inclusion and exclusion criteria. Inclusion criteria required snakes in good physical health without visible clinical signs suggestive of infection, with at least 72 h elapsed since the last feeding before handling, of appropriate size for safe handling and swabbing, absence of imminent shedding signs, and adequate hydration. Exclusion criteria eliminated gravid or breeding specimens, individuals with visible lesions or scarring, snakes displaying excessive stress or defensive behavior, recently acquired specimens in quarantine, and animals under medical treatment. These criteria were applied to safeguard animal welfare and ensure sample quality, consistent with captive reptile management recommendations. The present analysis focused on the oral cavity microbiota. Five venom specimens collected during the same sampling period were not included in the oral microbiota analyses and are described as excluded specimens below.

### 5.2. Study Population and Specimen Description

A total of 73 biological specimens were processed during the study period, comprising 68 oral swabs and five venom specimens collected from captive adult viperids representing four species: *Bothrops asper* (Ba), *Bothriechis nigroviridis* (Bn), *Cerrophidion sasai* (Cs), and *Porthidium lansbergii* (Pl) (Figure 7a–d). Each of the 68 oral swabs was collected from a different snake, and no snake contributed more than one oral sample to the analysis. The five venom specimens were excluded from the oral-community analysis because they represented a different biological matrix and were outside the scope of this study. All four species are native to Panama and distributed across lowland and premontane regions of the country; specimens were sourced from the CIIMET captive collection, which holds individuals of Panamanian provenance transferred from field collection sites across the national territory. All specimens were adults, housed individually in plastic rack enclosures under controlled environmental conditions with water provided ad libitum, regular cage disinfection, and a species- and size-tailored rodent diet, with adults fed every 20–30 days and prey mass corresponding to approximately 10–20% of body weight. All 68 oral samples met the predefined sequencing-depth criterion and were retained for downstream analyses (Table 1). Sample sizes varied across host species (*n* = 8–30), reflecting differential captive availability at the CIIMET serpentarium rather than a prespecified balanced design. This imbalance reduced precision for comparisons involving the smallest group (*B. nigroviridis*, *n* = 8) and is acknowledged as a study limitation.

### 5.3. Snake Handling and Sample Collection

Snake manipulation was performed exclusively by CIIMET personnel trained in venomous snake handling and herpetological techniques. Snakes were restrained behind the head to ensure safe control of the mouth and fangs while providing adequate body support and minimizing stress. Sedation was not used for adult *P. lansbergii*, *B. nigroviridis*, and *C. sasai*, as their smaller body size allowed direct, safe, and minimally invasive handling. Manual restraint enabled faster, more efficient procedures, reducing time outside enclosures and optimizing sampling with minimal animal impact. For *B. asper*, whose larger body size required additional restraint, CO_2_ sedation was performed using purpose-built containment chambers with CO_2_ supply systems. Individual specimens were maintained in pre-charged CO_2_ chambers for approximately 10–15 min to reduce reactivity and facilitate safe oral sampling.

Oral cavity samples were collected using sterile nylon swabs gently rubbed in the fang sheath and oral cavity to collect material from mucosal pockets and fangs. Swabs were immediately deposited in labeled liquid Amies transport medium (ESwab®; COPAN Italia S.p.A., Brescia, Italy).

### 5.4. Sample Transport and Storage

Oral swabs were placed immediately in liquid Amies transport medium (ESwab^®^) and transported to the laboratory according to the established CIIMET sample-handling protocol. Samples were subsequently stored at −80 °C until DNA extraction. Approximately 1 mL of transport medium was available per sample for downstream molecular processing.

### 5.5. Culture-Independent Bacterial Identification

Genomic DNA extraction from oral swab samples stored at −80 °C in liquid Amies transport medium (ESwab^®^) was performed using the ZymoBIOMICS DNA Miniprep Kit (Zymo Research, Irvine, CA, USA). This kit employs BashingBeads™ (Zymo Research, Irvine, CA, USA) for uniform mechanical lysis and OneStep™ technology for PCR inhibitor removal. DNA quality and concentration were assessed by spectrophotometry (Shimadzu Corporation, Kyoto, Japan), measuring A260/A280 ratios. For 16S rRNA gene amplification, the V3–V4 hypervariable region of the 16S rRNA gene was amplified using Illumina overhang adapter primers: 341F forward 5′-TCG TCG GCA GCG TCA GAT GTG TAT AAG AGA CAG CCT ACG GGN GGC WGC AG-3′ and 805R reverse 5′-GTC TCG TGG GCT CGG AGA TGT GTA TAA GAG ACA GGA CTA CHV GGG TAT CTA ATC C-3′, generating ~460 bp amplicons. This length refers to the amplified V3–V4 target. ASV inference and taxonomic assignment were performed using only the forward-read segment retained after primer removal and quality filtering, rather than the complete V3–V4 amplicon. PCR was performed in 25-µL reactions containing 2.5 µL of template DNA, 0.5 µL of each primer, 0.9 µL of MgSO_4_ (50 mM), 0.5 µL of dNTPs (10 mM), 2.5 µL of 10× buffer, 0.1 µL of Platinum Taq HiFi, and 17.5 µL of nuclease-free water. Cycling conditions were 94 °C for 2 min; 25 cycles of 94 °C for 20 s, 55 °C for 30 s, and 72 °C for 90 s; and a final extension at 72 °C for 6 min. Amplicons were purified with AMPure XP beads (1:1 ratio). Index PCR was performed using Nextera XT Index Kit (Illumina, Inc., San Diego, CA, USA) with unique dual-index combinations per sample: 12.5 μL of purified DNA, 2.5 μL of each index primer (N7xx and S5xx), and 7.5 μL of Nextera PCR Master Mix, amplified for 12 cycles (72 °C 3 min; 95 °C 10 s, 55 °C 30 s, 72 °C 30 s per cycle; 72 °C 5 min final). Libraries were quantified and manually normalized to a final concentration of 4 nM.

Indexed libraries were pooled (5 μL each), purified with AMPure XP beads (Beckman Coulter, Inc., Brea, CA, USA) (1.12x ratio), and diluted to 4 nM. Denaturation was performed by mixing 5 μL of pooled library with 5 μL of fresh 0.2 N NaOH for 5 min at room temperature, followed by the addition of 990 μL of pre-chilled Hyb buffer, yielding a 20 pM denatured library. Sequencing was performed on the Illumina MiSeq platform (Illumina, Inc., San Diego, CA, USA) using paired-end 2 × 250 bp chemistry, with a PhiX control spike-in (≥5%) for quality control and a final library concentration of 10 pM. Run quality was assessed as the proportion of bases with Phred quality scores ≥ Q30. A water no-template control was included in each PCR run as an amplification control. No band at the expected amplicon size was visible on a 2% agarose gel. The original workflow used the no-template controls for gel-based assessment only; they were not indexed or sequenced. Neither extraction blanks nor library-preparation blanks were included in the original experimental design. This omission was a limitation of the original experimental design. Absence of a visible band does not establish absence of contaminating bacterial DNA, and sequence-level assessment of reagent- or laboratory-derived contamination was therefore not possible using negative-control libraries.

### 5.6. Bioinformatic Processing and ASV Inference

Raw sequences targeting the V3–V4 hypervariable region were processed using DADA2 (v1.22.0) in R v4.3.2. Primer sequences were identified and removed using Cutadapt. Although sequencing was performed in paired-end mode (2 × 250 bp), only forward reads (R1) were retained for final ASV inference. After primer removal and quality filtering, the retained sequences were 233 nucleotides long. Taxonomic inference was therefore based on the retained forward-read segment rather than the complete approximately 460-bp V3–V4 amplicon.

The forward-read workflow was selected on the basis of read-retention diagnostics. In the paired-end assessment, approximately 60% of merged reads were removed during chimera filtering, and final retention was approximately 33% of input read pairs. The forward-read workflow retained approximately 84% of input R1 reads. These percentages describe processing losses and do not quantify the proportion of R2 reads affected by poor base-call quality. R2 reads were omitted from the final workflow to retain a larger proportion of the available sequence data. Sequences were denoised using a trained error model, and chimeras were identified and removed using removeBimeraDenovo. Taxonomic assignment was conducted against the SILVA v138.2 database.

Following restriction of the dataset to the 68 oral samples, the ASV feature table contained 3446 features. Mitochondrial and chloroplast-associated features were subsequently removed using the subset_taxa function in the phyloseq package v1.46.0, reducing the dataset to 3235 ASVs. A prevalence–abundance filter was then applied, retaining ASVs detected in at least 5% of samples with a minimum of 10 reads and yielding a final analytical dataset of 300 ASVs for downstream statistical analyses. Phylum-level summaries retain the lineage assignments used in the SILVA v138.2-based analysis. Under this classification, *Mycoplasma*-assigned ASVs were grouped within Bacillota. This grouping differs from the current LPSN placement of Mycoplasmataceae within Mycoplasmatota.

### 5.7. Exploratory Assessment of Potential Contamination

Sequenced extraction and library negative controls were unavailable; therefore, control-based identification of contaminant ASVs could not be performed [69,70]. Two exploratory assessments were conducted. First, the relative abundance of genera previously reported in reagent-contamination studies was summarized [70]. Such overlap was not considered proof of contamination, and genera were not automatically removed solely on this basis. Conversely, absence from the selected list was not interpreted as evidence of biological origin. Second, the association between sequencing depth and observed ASV richness was evaluated using Spearman correlation. This analysis assessed depth-related detection patterns and was not a contamination test. Neither assessment quantifies the contaminant contribution nor replaces sequenced negative controls. The prevalence–abundance filter restricts the analysed ASV set but cannot reliably distinguish contaminant sequences from biological sequences.

### 5.8. Rarefaction Depth Determination

Sequencing depth per sample was summarized per host species (Table 2). An outlier threshold was defined a priori as 10% of the median read count across all 68 retained samples (11,612 reads); no sample fell below this threshold. Rarefaction depth was therefore set to 49,273 reads/sample, the minimum sequencing depth observed among all 68 retained samples. This depth standardized sequencing effort for alpha-diversity comparisons while retaining all 68 samples in the analytical dataset [73]. Rarefaction does not eliminate contaminant sequences or recover ASVs removed during upstream filtering.

### 5.9. Compositional Statistical Framework and Visualization

Statistical analyses were selected according to the inferential objective and the properties of each data representation. Alpha diversity metrics (observed ASV richness, Shannon diversity, and Pielou’s evenness) were calculated using the microbiome package v1.24.0 after rarefying the prevalence–abundance-filtered ASV count table to 49,273 reads per sample. Observed ASV richness therefore represents the number of retained ASVs detected in each rarefied sample and should not be interpreted as the total bacterial richness of the oral community. Chao1 and ACE were not reported because denoising and subsequent prevalence–abundance filtering modify the low-frequency portion of the ASV distribution on which these estimators depend. Good’s coverage was reported descriptively. Between-species differences were assessed using the Kruskal–Wallis test, followed, when the global test was significant, by Dunn’s pairwise tests with Benjamini–Hochberg correction.

For beta-diversity analysis, genus-level count data were transformed using the centered log-ratio (CLR) transformation. Aitchison distances were calculated as Euclidean distances in CLR space. Differences in community composition among host species were assessed using PERMANOVA (adonis2, vegan v2.7-1) with 999 permutations. Homogeneity of multivariate dispersions was evaluated using betadisper; because the global PERMDISP test was significant, pairwise differences in distances to group centroids were examined using Tukey’s HSD test.

Differential abundance was evaluated separately using ANCOM-BC2 (v2.4.0), with host species as the grouping variable and a global test across the four species. *p*-values were adjusted using the Holm method to control the familywise error rate. Mean CLR-transformed genus abundances were used only for visualization in the differential-abundance heatmap; hierarchical clustering was performed using Ward’s minimum variance method (Ward.D2) on Euclidean distances.

## Figures and Tables

**Figure 1:**
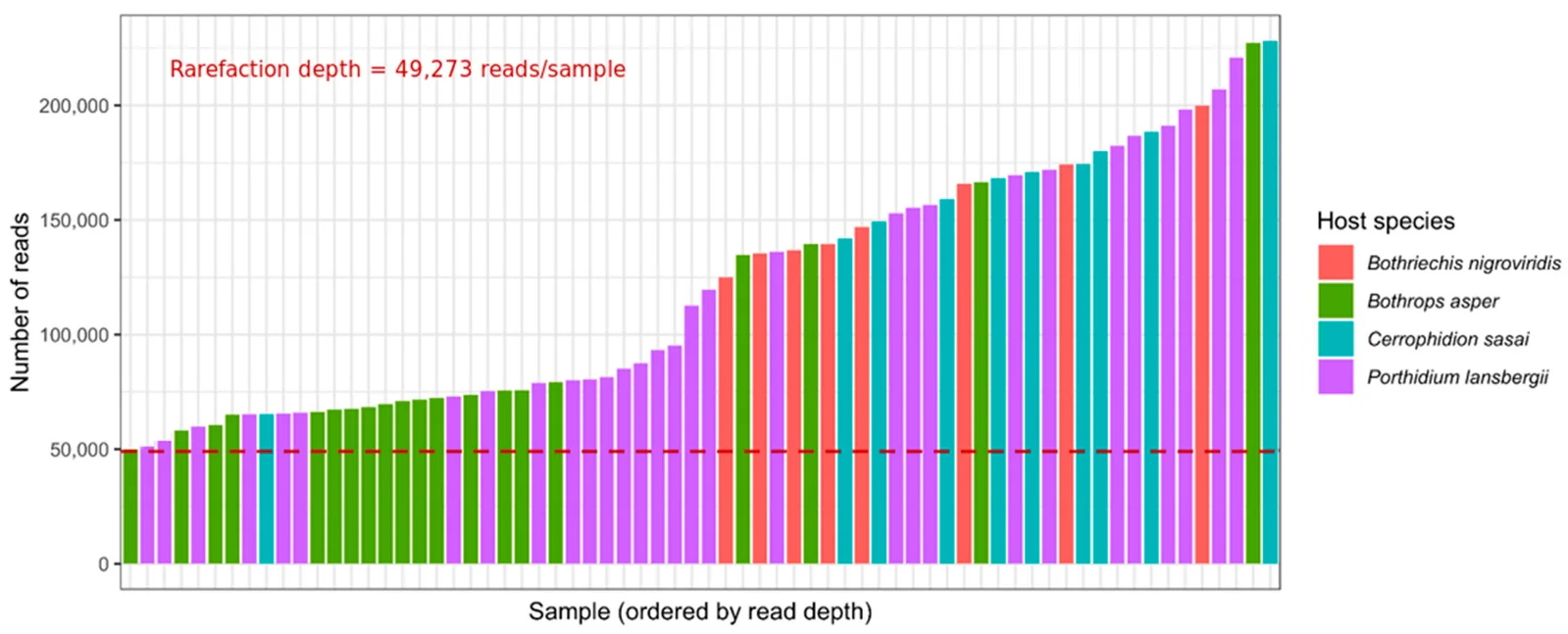
Sequencing depth distribution across oral microbiota samples (*n* = 68), ordered from lowest to highest read count and colored by host species. The dashed red line indicates the rarefaction depth (49,273 reads/sample). No sample fell below the predefined low-depth threshold (10% of the overall median read count; 11,612 reads); therefore, all 68 retained samples were included, and rarefaction depth was set to the minimum observed sequencing depth.

**Figure 2:**
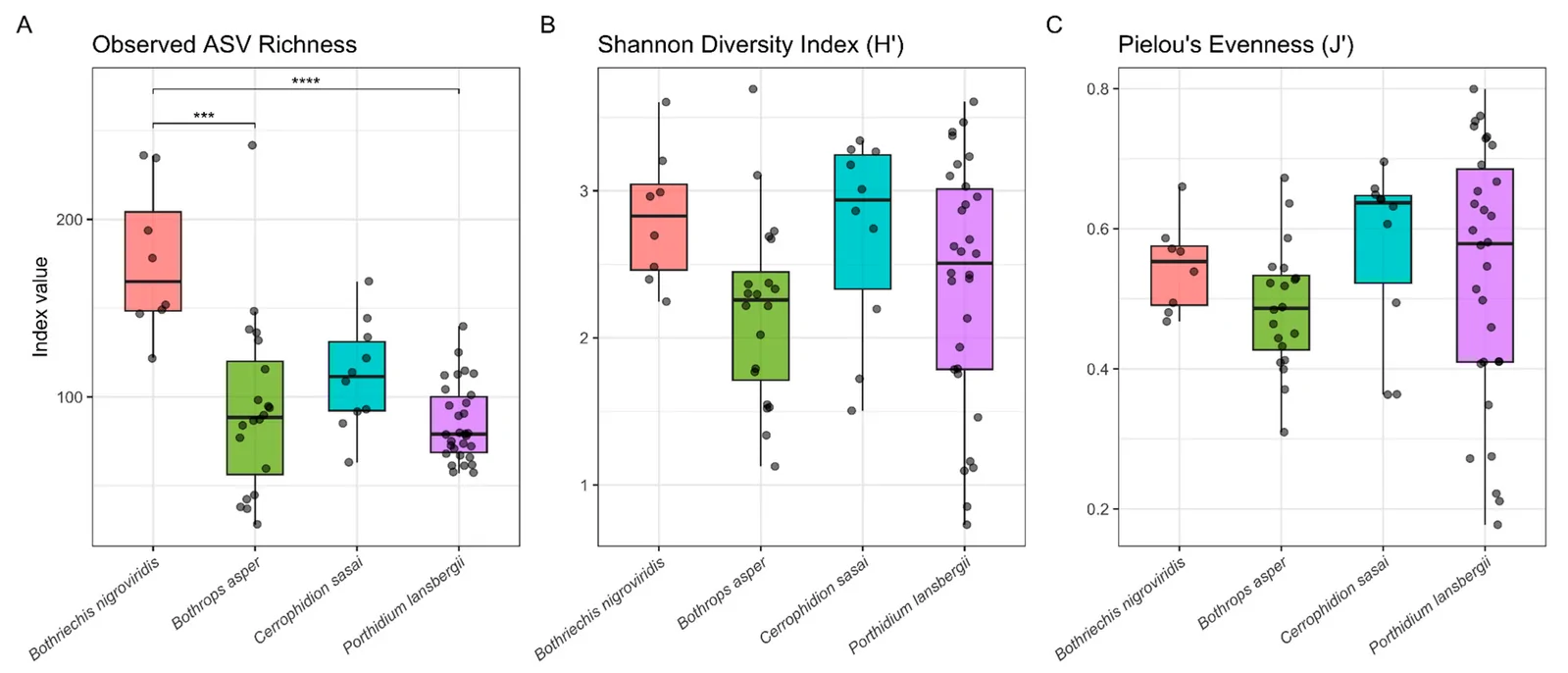
Alpha diversity of the oral microbiota across four viperid species (*n* = 68; rarefied to 49,273 reads/sample). Boxes represent the median and interquartile range, and individual points represent biological replicates. (**A**) Observed ASV richness differed significantly among species (Kruskal–Wallis, H = 21.87, df = 3, *p* < 0.001). Significant pairwise comparisons are indicated by brackets (Dunn’s test with Benjamini–Hochberg correction): *** adjusted *p* < 0.001; **** adjusted *p* < 0.0001. (**B**) Shannon diversity did not differ significantly among species (H = 7.07, df = 3, *p* = 0.070). (**C**) Pielou’s evenness did not differ significantly among species (H = 4.40, df = 3, *p* = 0.221).

**Figure 3:**
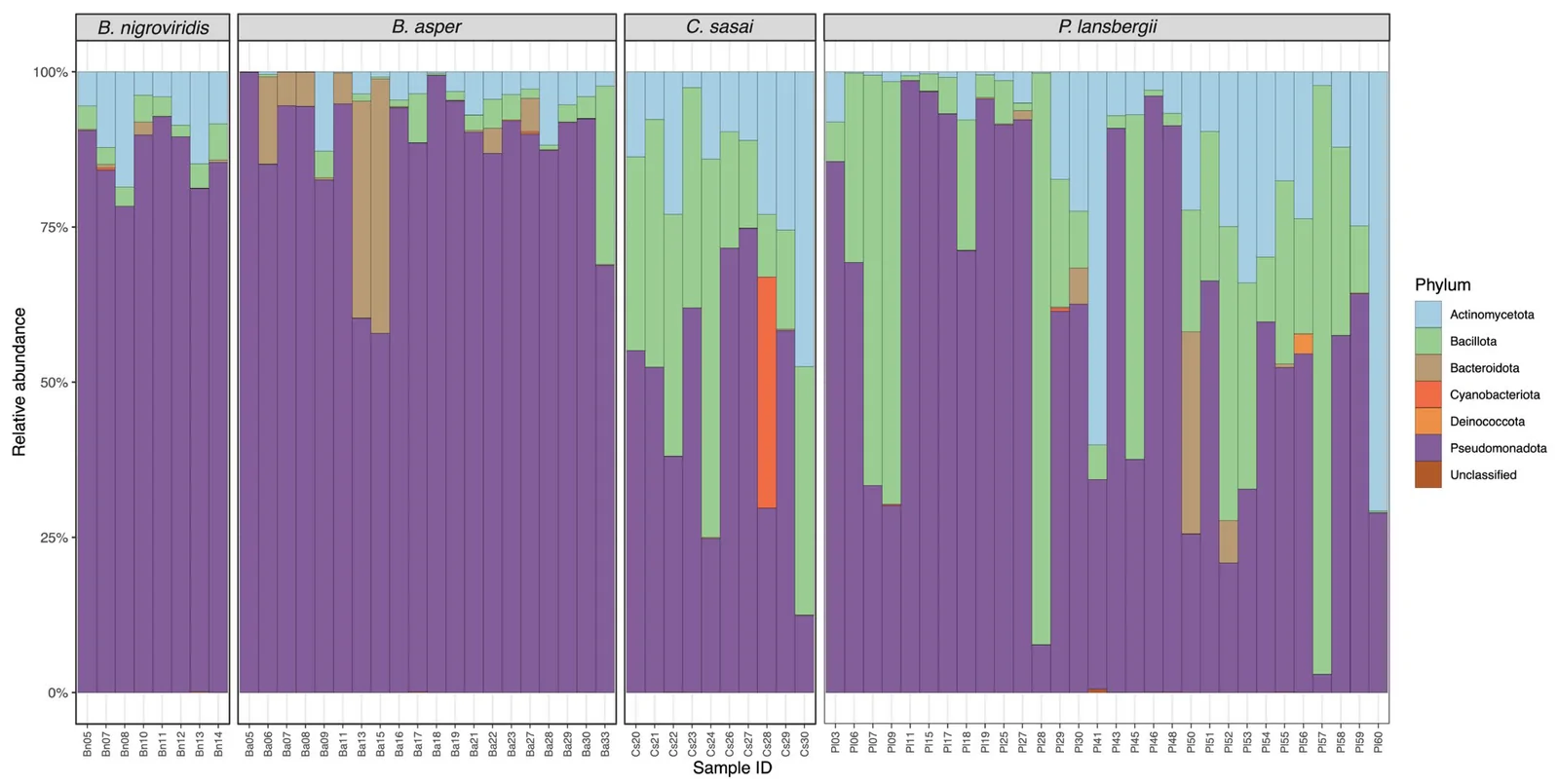
Taxonomic composition of the oral microbiota at the phylum level across all 68 retained samples. Each bar represents one sample; samples are grouped by host species (facets). Bars represent descriptive relative abundances prior to log-ratio transformation. CLR-transformed genus-level data were used for Aitchison-distance analyses, while differential abundance was evaluated separately using ANCOM-BC2. Communities were dominated by Pseudomonadota, with variable secondary contributions from Bacillota, Actinomycetota, and Bacteroidota. ASVs unassigned at the phylum level are labeled ‘Unclassified’. *Mycoplasma*-assigned ASVs are included within Bacillota according to the lineage used in the analysis; current LPSN taxonomy places Mycoplasmataceae within Mycoplasmatota.

**Figure 4:**
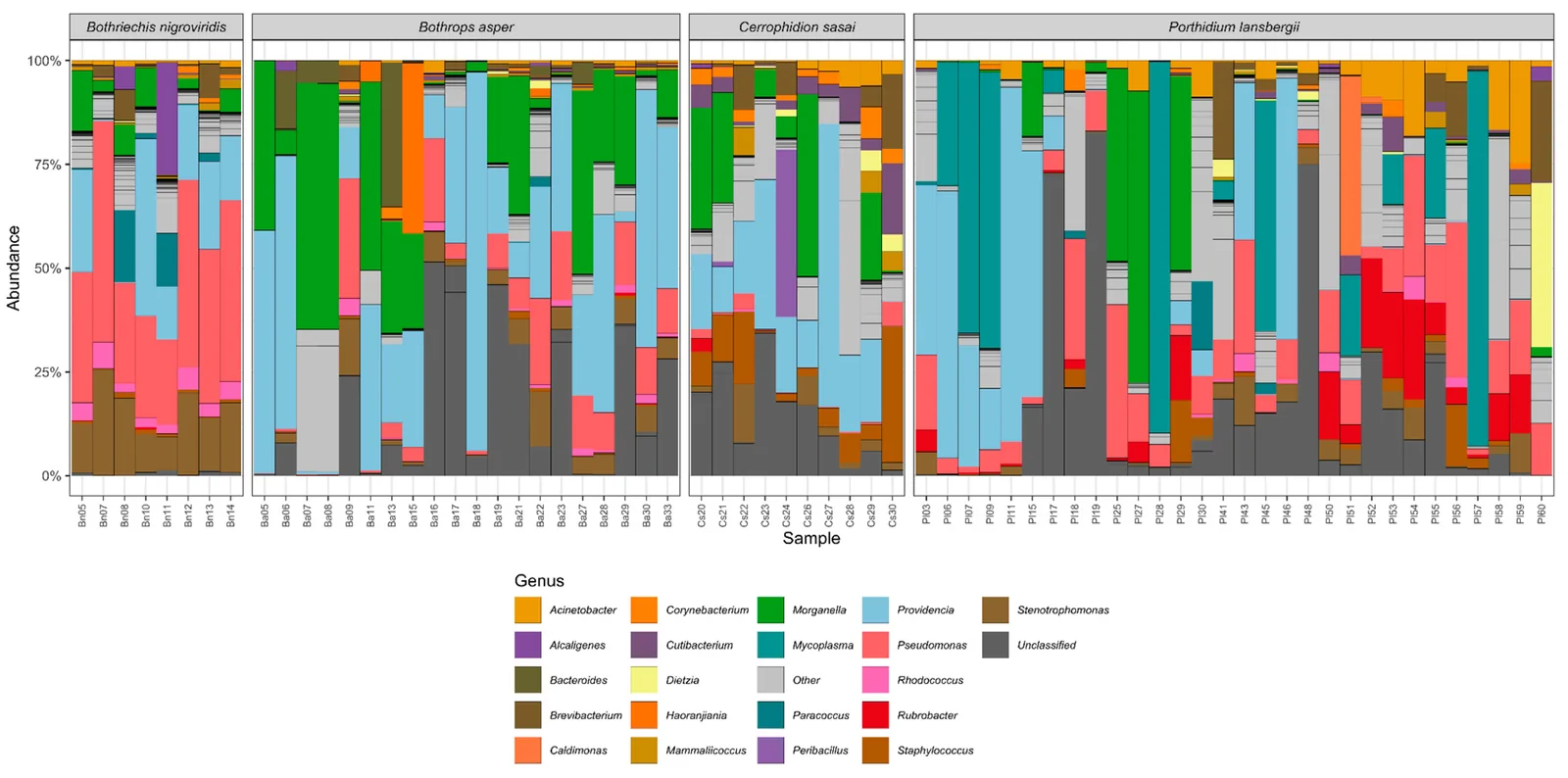
Relative abundance of the 20 most abundant bacterial genera in the oral microbiota across all 68 samples. Genera ranked 21st and below are collapsed into ‘Other’; ASVs without genus-level assignment are labeled ‘Unclassified’. Samples are grouped by host species (facets).

**Figure 5:**
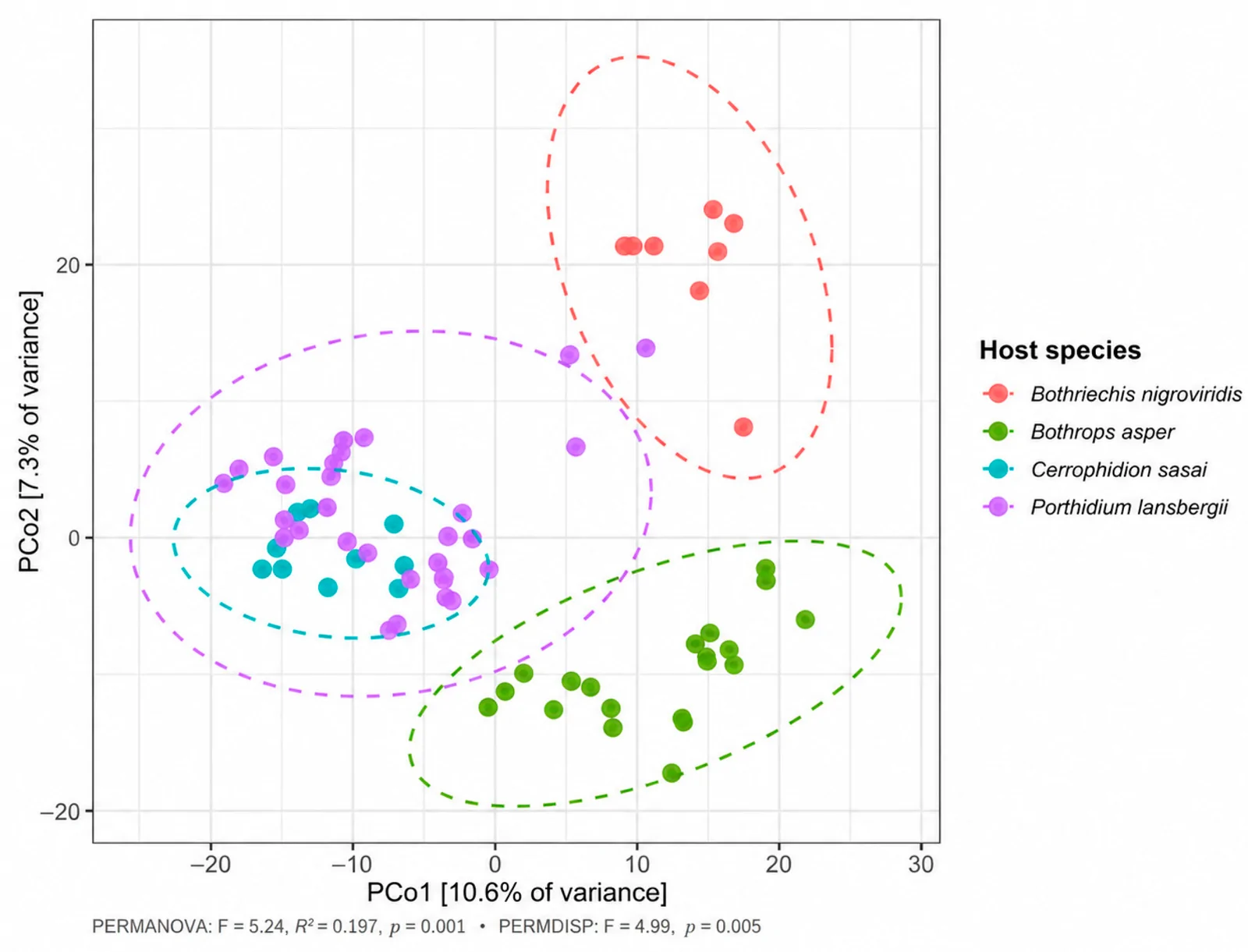
Principal coordinates analysis (PCoA) of Aitchison distances among oral microbiota samples (*n* = 68). Each point represents one sample; colors indicate host species. Dashed ellipses represent 95% data ellipses assuming a normal distribution within each group and are shown for visualization only. Axis labels indicate the percentage of total variance represented by each coordinate. Community composition was associated with host species by PERMANOVA (adonis2; 999 permutations: F = 5.24, R^2^ = 0.197, *p* = 0.001). PERMDISP indicated heterogeneity in within-group dispersion (F = 4.99, *p* = 0.005); therefore, apparent centroid separation should be interpreted alongside differences in within-group spread.

**Figure 6:**
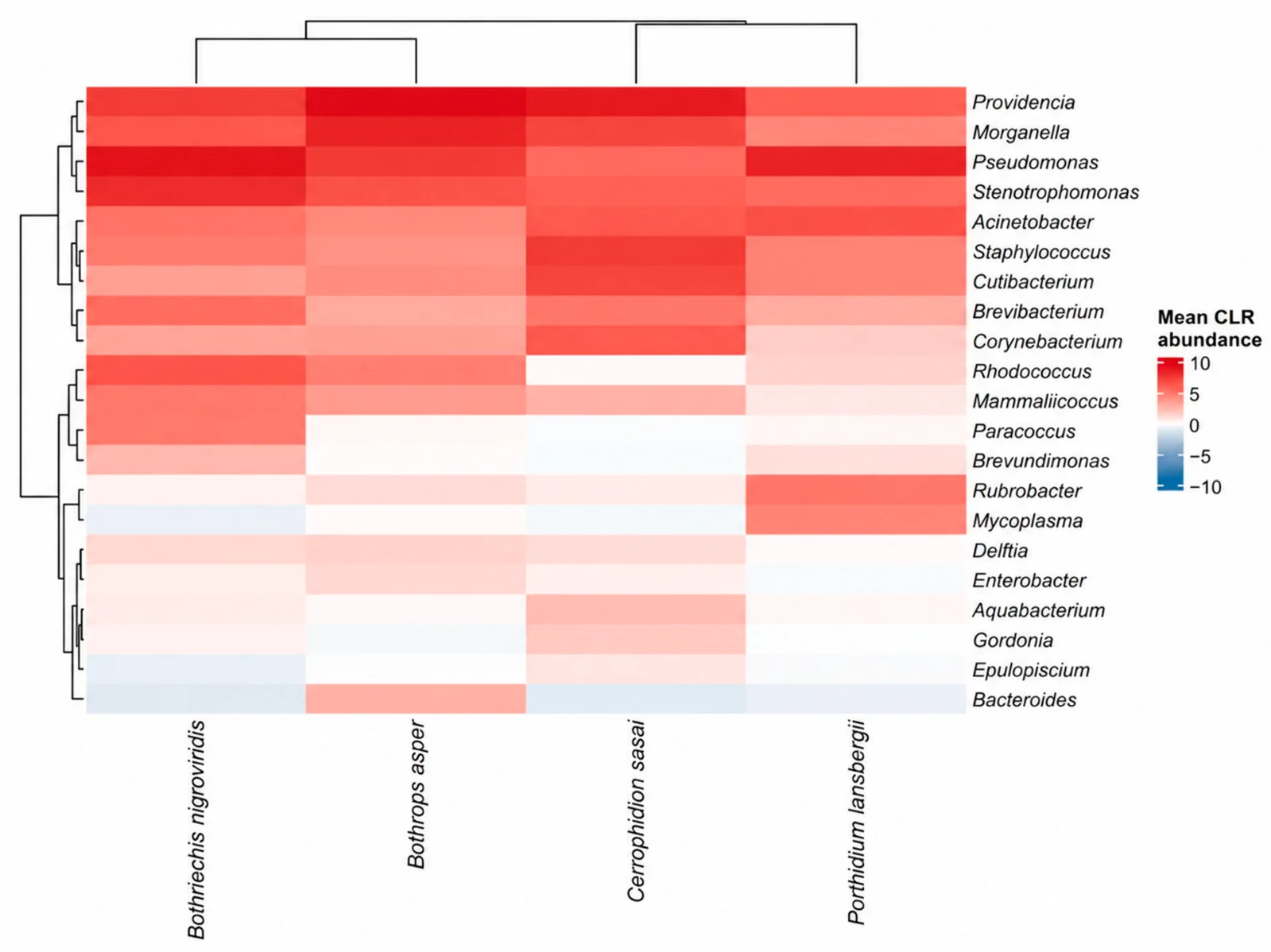
Heatmap of differentially abundant microbial genera in the oral cavity across four viperid species. Only genera identified as significantly differentially abundant by ANCOM-BC2 (global test, Holm–Bonferroni-adjusted q < 0.05) are shown. Colors represent mean CLR-transformed abundance per host species: blue indicates abundance below the compositional geometric mean; red indicates abundance above it. Both genera (rows) and host species (columns) were hierarchically clustered using Ward’s minimum variance method (Ward.D2) on Euclidean distances.

**Figure 7:**
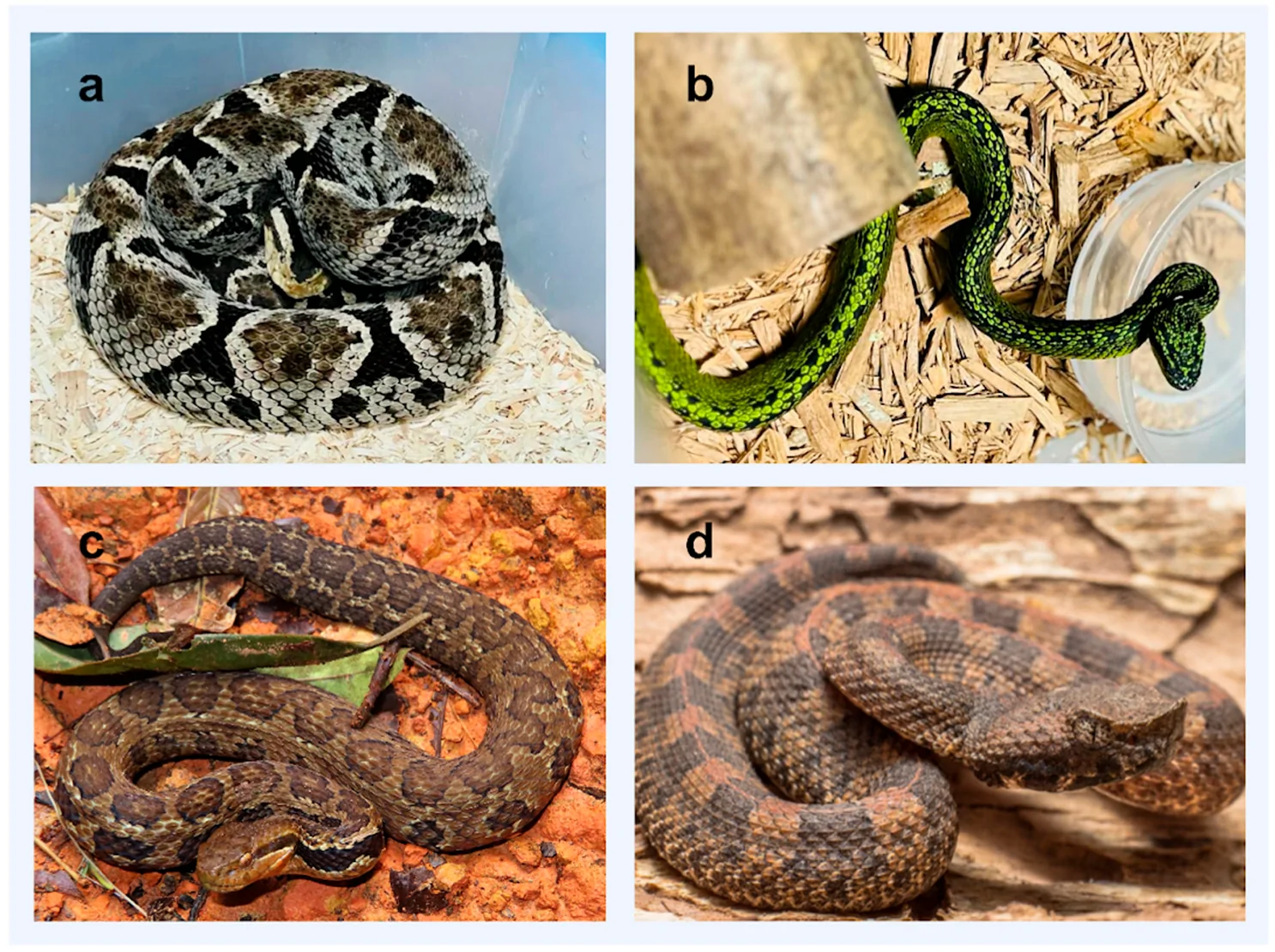
Representative images of the four viperid species included in this study: (**a**) *Bothrops asper* (*Ba*), (**b**) *Bothriechis nigroviridis* (*Bn*), (**c**) *Cerrophidion sasai* (*Cs*), and (**d**) *Porthidium lansbergii* (*Pl*). Photos courtesy of Stephany Young (**a**,**b**); images for (**c**,**d**) obtained from iNaturalist (https://www.inaturalist.org, accessed on 30 November 2025).

**Table 1:** Dataset composition throughout sample selection and ASV filtering. The initial imported dataset comprised 73 biological specimens (68 oral swabs and five venom specimens) and 3446 ASV features. Because the present study focused on the oral microbiota, the five venom specimens were excluded from downstream oral-community analyses, leaving 68 oral samples. The ASV feature-table dimension remained 3446 at this stage. Removal of mitochondrial and chloroplast-associated features reduced the dataset to 3235 ASVs. Subsequent prevalence–abundance filtering, retaining ASVs detected in ≥5% of samples with ≥10 reads, yielded a final analytical dataset of 300 ASVs across the 68 oral samples.

Dataset-Processing Step	Samples (*n*)	ASV Features (*n*)
Initial imported dataset	73	3446
Oral samples retained for analysis	68	3446
After organelle removal	68	3235
After prevalence–abundance filtering	68	300

**Table 2:** Sequencing depth summary per host species, including sample size (*n*), minimum, median, mean, and maximum read counts.

Host Species	*n*	Min	Median	Mean	Max
*B. nigroviridis*	8	125,030	143,282	152,996	199,856
*B. asper*	20	49,273	71,213	87,965	227,357
*C. sasai*	10	65,330	169,653	162,605	228,164
*P. lansbergii*	30	51,005	94,247	118,488	220,935

**Table 3:** Mean relative abundance (±SD, %) of the 20 most abundant bacterial genera in the oral microbiota per host snake species. The 20 genera were selected according to their overall abundance across all samples and are presented individually for descriptive comparison among host species. Genera ranked below the top 20 are not shown in the table. Values represent mean relative abundance (%) ± SD per host species. ASVs without genus-level taxonomic assignment are shown separately as “Unclassified”.

Genus	*Bothriechis nigroviridis*	*Bothrops asper*	*Cerrophidion sasai*	*Porthidium lansbergii*
*Acinetobacter*	0.99 ± 0.38	0.57 ± 0.69	2.31 ± 2.29	4.44 ± 6.02
*Alcaligenes*	4.09 ± 9.49	0.18 ± 0.55	0.16 ± 0.33	0.15 ± 0.65
*Bacteroides*	0.01 ± 0.02	3.37 ± 8.16	0.00 ± 0.00	0.00 ± 0.00
*Brevibacterium*	2.68 ± 3.32	0.55 ± 0.92	4.32 ± 6.17	2.57 ± 6.38
*Caldimonas*	0.00 ± 0.00	0.01 ± 0.03	0.05 ± 0.15	1.50 ± 7.89
*Corynebacterium*	0.47 ± 0.62	0.40 ± 0.73	2.62 ± 2.21	0.49 ± 1.19
*Cutibacterium*	0.19 ± 0.13	0.35 ± 0.37	4.83 ± 4.87	0.97 ± 1.82
*Dietzia*	0.07 ± 0.19	0.20 ± 0.47	1.18 ± 1.88	1.59 ± 7.22
*Haoranjiania*	0.00 ± 0.00	2.43 ± 9.15	0.00 ± 0.00	0.00 ± 0.00
*Mammaliicoccus*	0.99 ± 0.69	0.34 ± 0.32	1.70 ± 2.72	0.32 ± 0.86
*Morganella*	5.29 ± 5.03	21.32 ± 20.20	13.32 ± 15.72	6.32 ± 17.03
*Mycoplasma*	0.01 ± 0.01	0.01 ± 0.02	0.01 ± 0.01	15.35 ± 28.00
*Paracoccus*	4.27 ± 6.79	0.14 ± 0.54	0.01 ± 0.01	0.74 ± 3.04
*Peribacillus*	0.00 ± 0.01	0.00 ± 0.00	4.15 ± 12.72	0.00 ± 0.00
*Providencia*	16.95 ± 13.71	31.09 ± 24.46	21.94 ± 18.56	13.96 ± 24.50
*Pseudomonas*	35.01 ± 11.77	8.71 ± 8.29	1.40 ± 1.91	12.10 ± 10.30
*Rhodococcus*	3.78 ± 1.56	0.89 ± 1.09	0.08 ± 0.18	0.66 ± 1.50
*Rubrobacter*	0.10 ± 0.19	0.07 ± 0.17	0.33 ± 0.99	5.21 ± 7.55
*Staphylococcus*	0.73 ± 0.37	0.33 ± 0.45	8.92 ± 9.90	1.79 ± 3.79
*Stenotrophomonas*	15.31 ± 5.91	4.07 ± 4.04	3.09 ± 4.38	2.37 ± 3.08
Unclassified	0.13 ± 0.28	3.46 ± 10.30	2.87 ± 7.15	3.02 ± 11.62

## Data Availability

The sequencing data generated in this study have been deposited in the NCBI Sequence Read Archive (SRA) under BioProject accession PRJNA1436642.

## Supplementary Materials

The following supporting information can be downloaded at: https://www.mdpi.com/article/10.3390/toxins18100440/s1, Table S1a: Good’s coverage per host species; Table S1b: Kruskal-Wallis test results for alpha diversity metrics across host species; Table S1c: Post-hoc pairwise comparisons of observed ASV richness among the four host viperid species (Dunn’s test with Benjamini–Hochberg correction); Table S2: Genera with significant differential abundance across host species identified by the ANCOM-BC2 global test (Holm-adjusted q < 0.05); Table S3: Mean relative abundance (±SD, %) of bacterial phyla per host species; Table S4: Prevalence and relative-abundance distribution of Mycoplasma and Cutibacterium across host species.

## Abbreviations

The following abbreviations are used in this manuscript:

NGS	Next-Generation Sequencing
ASV	Amplicon Sequence Variant
DADA2	Divisive Amplicon Denoising Algorithm 2
PCoA	Principal Coordinates Analysis
PERMANOVA	Permutational Multivariate Analysis of Variance
PERMDISP	Permutational Analysis of Multivariate Dispersions
ANCOM-BC2	Analysis of Compositions of Microbiomes with Bias Correction 2
CLR	Centered Log-Ratio
FWER	Familywise Error Rate
Q30	Quality Score 30
R1/R2	Read 1/Read 2 (sequencing reads)
dNTPs	Deoxynucleotide Triphosphates
CLSI	Clinical and Laboratory Standards Institute
CIIMET	Centro de Investigación e Información de Medicamentos y Tóxicos
SILVA	SILVA ribosomal RNA database
Ba	*Bothrops asper*
Bn	*Bothriechis nigroviridis*
Cs	*Cerrophidion sasai*
Pl	*Porthidium lansbergii*
